# Structural insights into dynamics of RecU–HJ complex formation elucidates key role of NTR and stalk region toward formation of reactive state

**DOI:** 10.1093/nar/gkw1165

**Published:** 2016-11-28

**Authors:** Sagar Khavnekar, Sarath Chandra Dantu, Svetlana Sedelnikova, Sylvia Ayora, John Rafferty, Avinash Kale

**Affiliations:** 1 UM-DAE Centre for Excellence in Basic Science, University of Mumbai, Vidhyanagari Campus, Mumbai 400098, India; 2 Department of Biosciences and Bioengineering, Indian Institute of Technology Bombay, Powai, IIT Bombay, Mumbai 400076, India; 3 The Krebs Institute, Department of Molecular Biology and Biotechnology, University of Sheffield, Western Bank, Sheffield S10 2TN, UK; 4 Department of Microbial Biotechnology, Centro Nacional de Biotecnología, CNB-CSIC, 28049 Madrid, Spain

## Abstract

Holliday junction (HJ) resolving enzyme RecU is involved in DNA repair and recombination. We have determined the crystal structure of inactive mutant (D88N) of RecU from *Bacillus subtilis* in complex with a 12 base palindromic DNA fragment at a resolution of 3.2 Å. This structure shows the stalk region and the essential N-terminal region (NTR) previously unseen in our DNA unbound structure. The flexible nature of the NTR in solution was confirmed using SAXS. Thermofluor studies performed to assess the stability of RecU in complex with the arms of an HJ indicate that it confers stability. Further, we performed molecular dynamics (MD) simulations of wild type and an NTR deletion variant of RecU, with and without HJ. The NTR is observed to be highly flexible in simulations of the unbound RecU, in agreement with SAXS observations. These simulations revealed domain dynamics of RecU and their role in the formation of complex with HJ. The MD simulations also elucidate key roles of the NTR, stalk region, and breathing motion of RecU in the formation of the reactive state.

## INTRODUCTION

Cells have evolved efficient mechanisms to promote genetic diversity, ensure proper chromosome segregation and restore genome integrity via homologous recombination (1,2). This is an ubiquitous damage control process by which cells repair DNA and facilitate the restart of the stalled replication (1,3–8). This process may involve the formation of a four-way DNA crossover known as a Holliday junction (HJ) from the pairing of homologous duplex DNA. DNA recombination has been extensively studied in Gram negative bacteria such as *Escherichia coli* in terms of the genetics and the molecular machinery (9). *In vivo* the HJ is formed by the regression of a stalled fork by the action of *E. coli* RecG and may be translocated through thousands of base pairs by the RuvAB translocase complex (10). Branch migration enables the positioning at the junction of HJ intermediates of target sequences for subsequent cleavage by the RuvC HJ resolving enzyme (HJR), allowing for the separation of paired duplex DNA and normal chromosome segregation at cell division (11).

Our understanding of how the various components interact to establish and complete the homologous recombination reaction is less clear in Gram-positive bacteria, as there are marked differences in some components of the process. RecU HJ resolving enzymes are present in all Firmicutes, but they are not present in Gram-positive bacteria with high dG+dC content. These bacteria have RuvC homologues. RecU is involved in DNA repair and recombination (3–8,10) Biochemical data have shown the interaction between the RecU enzyme and the RuvAB complex (12), which is in agreement with the genetics data which show that *recU* and *ruvAB* share common suppressors (6). However, there are other genetic studies that suggest that RecU may participate together with RecG in some resolution reactions (10).

Structural characterization studies of HJ resolvases has classified them into two principal folds (12), with the exception of *E. coli* RusA (13) and bacteriophage T4 endonuclease VII (14). The first group resembles restriction endonucleases and includes archaeal Hjc and Hje resolvases (15,16), as well as bacteriophage T7 endonuclease I (17) and the second group, resembles HIV integrase and includes *E. coli* RuvC (18) and *Schizosaccharomyces pombe* Ydc2 (19). RecU belongs to the first group but has an additional central stalk region not seen in other family members (20).

We have previously reported the structure of RecU from *Bacillus subtilis* (20), which revealed the overall shape and details of the likely active sites in the dimer. RecU has a mushroom-like appearance with a cap (residues 34–55 and 90–199) and stalk sections (residues 56–89) (20,21). However, this structure lacked information on the N-terminal 1–33 residues (NTR) and the stalk region, which emerges from the centre of the dimer interface. The missing ‘mushroom stalk’ could be clearly seen in the structure of a homologous RecU from *Bacillus stearothermophilus* (PDB entry 1Y1O) but the NTR region remained elusive (22). Both regions are essential for RecU activity. Biochemical data showed that the stalk region of the RecU resolvase is essential for HJ recognition and distortion (4). It is also involved in interaction with RecA (23). Furthermore, once HJ is bound, RecU fails to modulate RecA activities, suggesting that both interactions are exclusive (4). Binding of ΔNTR-RecU (deleted residues 1–32) to HJ is very unstable, and that is why this mutant does not cleave the HJ properly (24).

In this work, we report the first crystal structure of catalytically inactive RecU (RecU_D88N_) bound to a 12 base pair palindromic dsDNA fragment, with well-defined NTR and stalk regions. The crystal structure of this protein–dsDNA complex provides insights into the binding of the DNA to RecU and the organization of its phosphate backbone relative to the active site and the stalk region. We also present the solution structure analysis of unbound RecU using SAXS, which confirms the conformational variability of the NTR region elusive from the previously reported X-ray structures. A thermofluor-based assay was carried out to test the induced stabilization of the purified protein (RecU_D88N_) against HJs of varying lengths. To gain insights into the conformational properties of RecU and its complex, we have used Molecular Dynamics (MD) simulations of wild type RecU (RecU_WT_), wild type RecU in complex with HJ (RecU_HJ_), ΔNTR mutant (residues 34–199) RecU (RecU_ΔNTR_) and ΔNTR mutant RecU in complex with HJ (RecU_ΔNTR-HJ_). These simulations allowed us to propose how the conformational dynamics of mushroom domain with respect to the stalk region can be used to bind to the HJ. Further, NTR region might have a role in the stabilization of the reactive state during the reaction mechanism.

## MATERIALS AND METHODS

### Protein synthesis and crystallization

Catalytically inactive mutant of RecU_D88N_, which binds but doesn't cleave HJ, was purified as described previously (4). The 12 base palindromic DNA fragment (ACGCAATTGCGT) was chemically synthesized (Yorkshire Bioscience, York, UK). The synthesized DNA was dissolved to make a stock solution of 1 mM in autoclaved water and was heated at 95°C for 5 min in a beaker of water where it was left to anneal overnight in an insulated flask. The purified protein was concentrated to 10 mg/ml (as estimated by Bradford assay) and RecU–DNA complex was formed by mixing the protein and the DNA to 1:1.25 molar ratio and incubating for 5 h at room temperature prior to crystallization. Crystals were obtained in sitting drop trials after two weeks at 17°C in a condition composed of 0.1 M BICINE pH 9, 10% PEG 6000.

### Data collection and refinement

Diffraction data for the RecU–DNA complex crystals were measured with synchrotron radiation at the Diamond Light Source, Oxford, United Kingdom on I02 beamline. Before data collection, crystals were transferred into a cryo-protecting solution composed of 0.1 M BICINE pH 9, 10% PEG 6000, 30% ethylene glycol and were further incubated for 30 min at room temperature, before flash cooling in liquid nitrogen. The data were processed with XDS (25) and merged using Scala (26) as implemented in the CCP4 software suite (27). The relevant data statistics are provided in Table 1.

**Table 1.** Data statistics for processing and refinement of diffraction data are shown

Data statistics	
Wavelength (Å)	0.9
Resolution range (Å)	48.7–3.2 (3.3–3.2)
Space group	R 3 2:H
Unit cell	144.4 144.4 310.3 90 90 120
Total reflections	226725 (22642)
Unique reflections	20730 (2042)
Multiplicity	10.9 (11.1)
Completeness (%)	99.8 (99.2)
Mean *I*/sigma(*I*)	19.9 (1.6)
Wilson *B*-factor	118.3
*R*-merge	0.09081 (1.596)
*R*-meas	0.0954
**Refinement statistics**	
*R*-work	0.2561 (0.3267)
*R*-free	0.3286 (0.3908)
RMS (bonds)	0.012
RMS (angles)	2.10
Ramachandran favored (%)	84
Ramachandran generously allowed (%)	14.4
Ramachandran outliers (%)	1.6
Clash score	29.4
Molprobity	2.8

Data in parentheses correspond to the highest resolution shell.

(1) *R*_merge_ = ∑ | *I* – <*I*> | / ∑ *I*, where *I* is the observed intensity and <*I*> the average intensity of reflections.

(2) *R*-factor = ∑ | *F*_obs_ – *F*_calc_ | / ∑ *F*_obs_.

Data in parentheses correspond to the highest resolution shell. The table was generated using the Phenix table utility.

The crystal diffracted to 3.2 Å resolution and belonged to space group *R*32. Error in the coordinates (0.9 Å) was calculated over all the four molecules of the RecU present in the ASU. Solvent content of 56% was calculated using the program Matthews (28,29). An initial set of phases were obtained by the molecular replacement method using the program Phaser (30) within the PHENIX software suite (31) using the structure of *B. subtilis* RecU (20) (PDB id: 1ZP7) as a search model. Several iterations of manual building using the program COOT (32) were alternated with cartesian and real space refinement using the program Phenix. Refine (33,34). Secondary structure restraints were imposed during refinements owing to low resolution data. Atomic B-factors were refined and were treated as isotropic. NTR atoms were refined for partial occupancies. A model of the flexible NTR region was validated by calculating composite omit maps (35). To further support the model of the NTR, we calculated crystal contacts made by the NTR within the crystals using NCONT in the CCP4 software suite. The model quality was validated for all structures using COOT and MolProbity (36). The statistics of the refined structure are given in Table 1. Structure was analyzed using COOT and PyMol (37). PyMol, VMD (38) and Ligplot+ (39,40) were used to visualize and interpret the interactions and produce figures. The coordinates of this structure has been deposited in Protein Data Bank (PDB) under the accession id: 5FDK.

### SAXS measurements

SAXS data were acquired at the ID14-3 Bio-SAXS beamline at ESRF, Grenoble, France. The typical beam flux was 2 × 10^12^ photons/s and the size of beam at the detector was 140 × 80 μm^2^. X-ray wavelength was selected using the monochromator to be 0.1 nm. For SAXS measurements, a CCD detector with a pixel size of 63 μm/pixel and a total size of 1344 × 1024 pixels was used to measure the scattered radiation. The detector was placed behind a vacuum path and the camera length was 1.7 m. The data from SAXS measurements were processed and analyzed using ATSAS (41).The data were scaled averaged and merged using PRIMUS (42). Analysis of the data was carried out with GNOM (43). Ensemble optimized modeling (EOM) was performed for the flexible N-terminal region (33 residues) with EOM 2.0 (44,45). For the EOM, the structure of a RecU dimer without the 33-residue NTR was given as an input and was treated as the core rigid structure. C_α_ atoms of these 33 residues were modeled based on the SAXS data with P2 symmetry restrictions.

### Thermoflour assay

All the DNA constructs were ordered from Eurofins, Bangalore, India. Different forms of duplexes and HJs were prepared by combining different mixtures of oligonucleotides (Supplementary Table ST1 and Figure S1) at 100°C in autoclaved nuclease free water to ensure melting of base pairing and then allowing a mixture to anneal overnight in a thermo-jacketed flask prefilled with boiling water. The formation of higher oligomeric species (duplex, or four-way junctions) for these constructs were qualitatively checked using a 2% agarose gel (Supplementary Figure S2). Different DNA constructs and the purified RecU_D88N_ were mixed in 1:1 molar ratio. The concentration of RecU_D88N_ was 5 μg per reaction well in a purification buffer (100 mM MES pH 6.5). Thermofluor assays were carried out using Sypro orange dye (Invitrogen, Bangalore, India) with a BIO-RAD CFX96 Touch RT PCR machine with a linear temperature gradient from 25°C to 100°C using 1°C step increments. The data, averaged over four experiments, were analyzed using Origin (OriginLab, Northampton, MA, USA) where the curves were smoothened using five points averaging and melting points (*T*_m_) were obtained by taking the first derivative of the respective curves. To analyze the stabilization of RecU on binding to HJ we calculated the conformational entropy for control (RecU_D88N_) and RecU_D88N_+HJ24 as follows.

\begin{equation*}\Delta {{H}} - {{T}}\Delta {{S}} = - {{RT}}\;\ln \;{{{K}}_{{\rm{eq}}}}\end{equation*}

\begin{equation*}\ln{K_{\rm eq}} = \left( {\frac{{ - \Delta H}}{R}} \right)\ \frac{1}{T} + \frac{{\Delta S}}{R}\end{equation*}

\begin{equation*}\ln{K_{\rm eq}} = \frac{{[\rm unfolded]}}{{[\rm folded]}}\ \end{equation*}

Δ*H* = -*Rx* slope of $\ln{K_{\rm eq}}$ versus 1/*T* graph

\begin{equation*}{\rm Conformational}\;{\rm entropy} = \Delta S = \frac{{\Delta H}}{{{T_{\rm m}}}}\end{equation*}

### Molecular dynamics simulations

#### Modeling of the starting structure

To create the starting structure of RecU with the HJ for the molecular dynamics (MD) simulations we initially created the RecU dimer. Of the four polypeptide chains in the asymmetric unit (A–D), chain D was modeled completely and we used it as the template to create the RecU dimer. Mg^2+^ ions were added based on their locations in the structure of *B. stearothermophilus* RecU (PDB entry: 2FCO) (21). All the side chains were included in the model. An HJ model was created in COOT using the two DNA duplexes seen in the protein–DNA structure presented here. A copy of the two duplexes present in the X-ray structure was rotated by 90^o^ around the two fold symmetry axis of the RecU dimer. The four duplexes (two originals and two copies) were now treated as the four arms of an HJ and the duplex copies were then rotated around their helical axes to match the 5′ and 3′ ends of the strands in each adjacent arms. The ends were ligated at the point of crossover. The N-terminal region was used as a guide to dock the two rotated arms (see Supplementary Figure S3 for more details). Finally, any steric clashes were removed with rigid body refinement. A starting structure for the ΔNTR variant simulations was created by deleting residues 1–33 of the NTR region from each monomer.

### Simulation setup

Using RecU dimer and the modeled HJ, we created the starting structures for the MD simulations of wild type RecU (RecU_WT_), wild type RecU in complex with HJ (RecU_HJ_), ΔNTR variant RecU (RecU_ΔNTR_), and ΔNTR variant RecU in complex with HJ (RecU_ΔNTR-HJ_). All our simulations were performed using the GROMACS package version 4.6.4 (46–48). Amber14sb (49) force field for the protein and parmbsc0 forcefield (50) for the DNA were used in all our simulations. TIP3P water model (51) was used and ions were described using parameters from Joung *et al.* (52). Each protein system was placed in a dodecahedron box with a minimum distance of 1 nm to the box walls. Every simulation box was solvated and the system was neutralized by adding 150 mM NaCl ions. Periodic boundary conditions were applied in all the directions. Each system was energy minimized using a steepest descent algorithm until the largest force acting on the system was smaller than 1000 kJ/(mol nm). The final structure from energy minimization was subjected to temperature equilibration using the Berendsen thermostat (53) to 298 K using a tau-t of 0.1 ps in 100 ps, followed by isotropic pressure equilibration to 1 atm using the Berendsen barostat (54) in 1 ns using a tau-p of 1 ps. During both the equilibration stages, main chain atoms were position restrained using a force constant of 1000 kJ/(mol nm^2^) in all directions. The final pressure equilibrated structure was used for production run simulation during which the temperature was maintained at 298 K using a velocity re-scaling thermostat (55) (tau-t: 1 ps) and Parrinello-Rahman barostat (56) (tau-p: 2 ps). Parallel LINCS (57,58) algorithm was used to constrain all bonds and electrostatics were treated using a Particle mesh Ewald scheme (59,60). Cut-off of 1 nm was used for electrostatics and a short range van der Waals cut-off of 1 nm was used. For each system, three independent 250 ns long production run simulations were carried out. Snapshots were saved at every 40 ps of the respective trajectories. Analysis tools within GROMACS and in-house Python code was used to analyze MD trajectories. Pymol and VMD were used to visualize the trajectories and render the images. All our simulation runs were carried out using the PARAM Yuva-II supercomputer at the National Param Supercomputing Facility (NPSF) at CDAC, Pune, India.

## RESULTS

### Structure of the RecU–DNA complex

**Figure 1.**
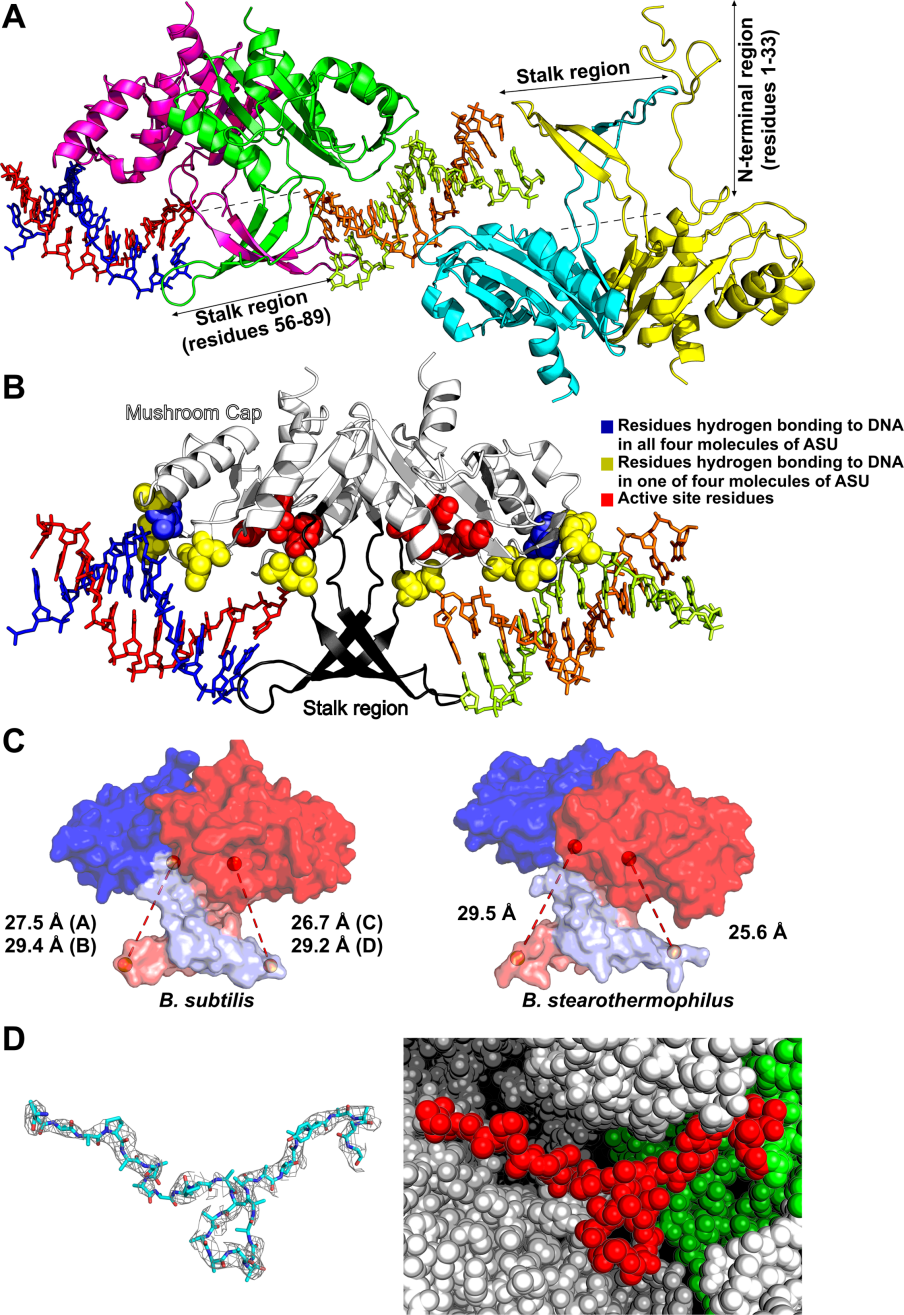
Crystal structure of inactive mutant of RecU (RecU_D88N_)–DNA complex. (**A**) Crystallographic asymmetric unit (ASU) in crystal structure of RecU–DNA complex consists of four RecU monomers forming two dimers bound to two DNA duplexes. Cartoon representation of protein Chains A, B, C and D are shown in green, cyan, pink and yellow respectively. Chain E, F, G and H of DNA are shown in orange, limon, blue and red respectively. (**B**) Interactions of RecU with DNA duplexes: Cap and stalk regions are shown in white and black, respectively. Hydrogen bonding and active site residues are shown as van der walls spheres, while the DNA is shown as stick. Residues which form hydrogen bonds in all four monomers of RecU in ASU are shown in blue. Residues that form hydrogen bonds in only some of monomers are shown in yellow. Active site residues are shown in Red. (**C**) Distance between Cα atoms of residues R71 of one monomer and D88 of its dimerising partner (binding pocket) for both AC and BD dimer are shown for the structure presented here and for the structure of *B. stearothermophilus*. (**D**) Electron density (2*F*_o_ – *F*_c_ map) is shown at sigma level of 0.7 for the 33 residue long NTR region of chain D. the sphere representation is shown for the NTR coloured red. The ASU is coloured green while symmetry mates forming the crystal contacts are coloured white.

Currently there are no structural data available of RecU in complex with HJ. Our attempts to crystallize RecU in complex with HJ (variable lengths/sequences/forms) were unsuccessful. Therefore to gain insights into RecU DNA interactions, we crystallized an inactive mutant RecU_D88N_ in complex with a palindromic 12 bp DNA. The asymmetric unit (ASU) of the crystal structure presented here consists of four RecU monomers (two dimers) and two dsDNA molecules (Figure 1). The two protein dimers are constituted by chains A plus C and B plus D and the two DNA duplexes are constituted by chains E plus F and G plus H. Each RecU monomer displays an α/β architecture and the structure of the protein is identical to the previous structure of unbound wildtype RecU (21) with no indications at this resolution of any kind of large conformational change. The backbone Root Mean Square Deviation (RMSD) between the previously reported structure (PDB ID: 1ZP7) and both the A/C and B/D dimers reported here is 0.95 Å. The complete stalk region of RecU is visible in both dimers within the ASU of our complex structure. The 33 residue long NTR could be completely modeled for chain D. For chains A and C, the NTR could be modeled from residue 23 and 26 onwards, respectively, whereas for chain B we could not model any of the NTR residues. The electron density and crystal environment for this 33 amino acid long region in chain D is shown in Figure 1D. Density for this region in chain-D is also seen in the composite omit map (see Supplementary Figure S4). NTR modeled in chain D shows a total of 76 crystal contacts as calculated using a cut-off distance of 5 Å.

### RecU–DNA interactions

The E/F and G/H DNA duplexes flank the stalk region of the A/C dimer (see Figure 1). Duplexes fit along the concave surface of the mushroom cap of each monomer, which also contains the active site pockets. The E/F DNA duplex is sandwiched between the A/C and B/D dimers. The G/H DNA duplex is sandwiched between A/C and a symmetry related (X+1/3, Y+2/3, Z+2/3) B/D dimer.

Analysis of our complex structure has revealed interactions between the protein and the DNA (Figure 1B). DNA is observed to be bound through a combination of hydrogen bonds (Supplementary table ST2) and long range interactions. A total of 14 protein–DNA hydrogen bonds can be proposed in the existing structure. This number is likely to be a lower estimate of the total number because many side chains could not be modeled fully. However, even at the low resolution, two hydrogen bonds are observed in all four monomers of the ASU: between the N atom of K165 and O1P of the nucleotide backbone; and between the OG atom of S166 and O2P of the nucleotide backbone. The number of hydrogen bonds with DNA per chain A, B, C and D are 5, 4, 2 and 3, respectively.

Apart from these hydrogen bonding interactions, long range interactions were also calculated using PDBSum (61). We could observe a total of 174 long range interactions (cut-off distance of 5.0 Å) between protein and the DNA. Of these, chain A has 49, chain B has 35, chain C has 30 and chain D has 60 non-bonded contacts with DNA. Though the density for the side chains of F81 and Y68 is not seen, they are well positioned to stabilize the approaching DNA by stacking against the nucleobases at the point of HJ crossover. The phosphate atom of the nearest base of the DNA is at a distance of ∼11 Å from the Cα atom of mutated active site residue N88D. Further, in the current study, we could not identify the position of Mg^2+^ atoms in the active site.

### Flexible stalk

The stalk consists of a β-hairpin of class 12:12 in each monomer. It comprises a terminal loop (residues 66–77) and two anti-parallel beta sheets (residues 61–65 and 78–81). The β-hairpins from the monomers cross over and extend toward the DNA duplex bound to the body of the other monomer in the dimer (Figure 1A). Hence, the DNA binding pocket consists of a mushroom cap of one monomer and a stalk of a dimerizing partner. Turn region of the β-hairpin consist of residues K70 and R71. This turn region is positioned symmetrically with respect to the mushroom cap such that the distance measured between Cα atoms of residues R71 of one monomer and N88 of its dimerising partner (binding pocket) is the same for both pockets of the dimer. This distance is 26 Å for the AC dimer and 29 Å for the BD dimer.

We compared this arrangement of stalk region with that of the previously reported DNA-unbound structure of *B. stearothermophilus* RecU. Its sequence has 57.6% identity and 73.3% similarity with that of *B. subtilis* RecU as observed from global alignment performed using Needle^51^. Further, the stalk region of RecU in both these organisms is completely conserved. In contrast to our structure where we observe a symmetric arrangement, distances between Cα atoms of corresponding residues R72 of one monomer and D89 of its dimerising partner were observed to be 29.5 Å and 25.6 Å, indicating an asymmetric arrangement of stalk region with respect to the mushroom cap (Figure 1C).

### SAXS

**Figure 2.**
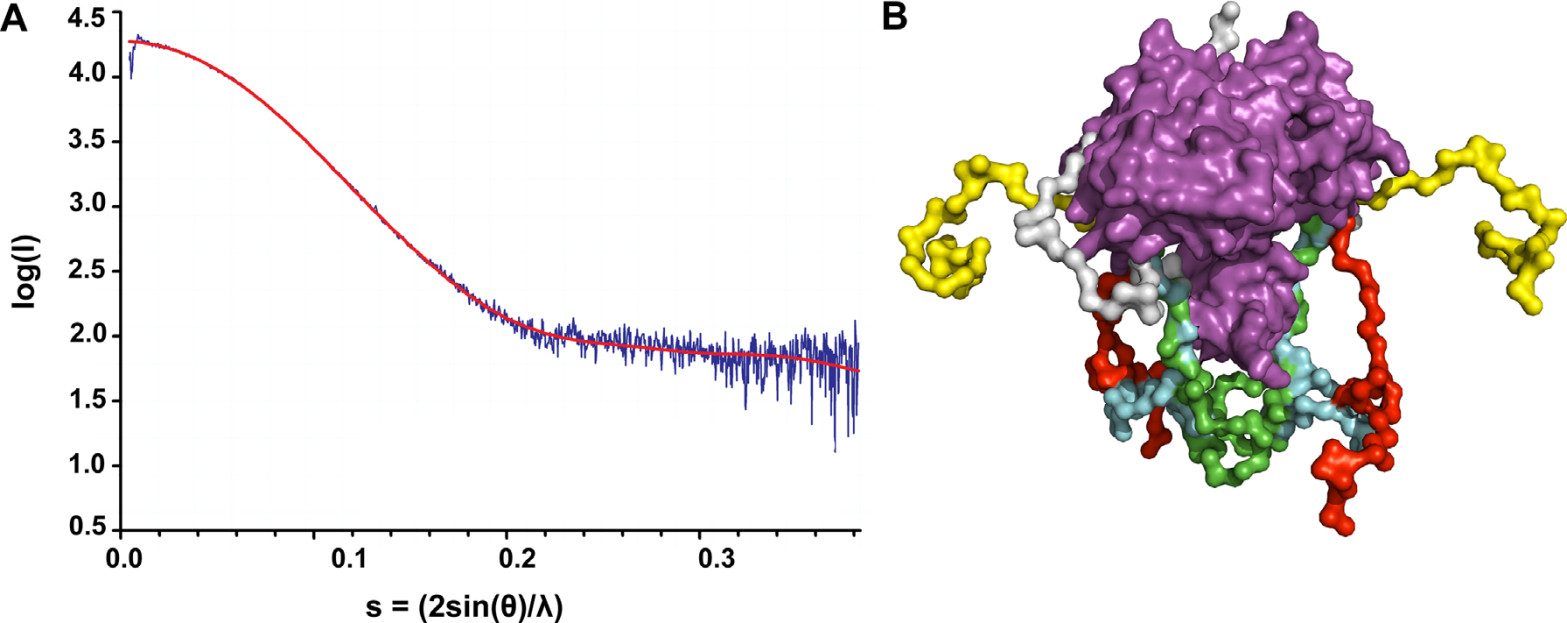
SAXS analysis of DNA free RecU (D88N). (**A**) Fit of Ensemble Optimized Modeling (shown in red) of SAXS data to the experimental SAXS data (shown in blue). The χ^2^-value of the fit is 1.89. (**B**) Ensemble of conformations of NTR generated from EOM analysis are shown in different colors. The core structure of the RecU enzyme is shown in magenta. Completely extended conformation of NTR are shown in yellow and red, partially collapsed conformation in green and cyan, and completely collapsed conformation of NTR is shown in white.

The poor electron densities of the NTR regions in the X-ray data imply that this region might be conformationally plastic. So, we performed SAXS experiments on the RecU_D88N_ in solution. EOM modeled data could be fitted onto the experimental SAXS data with a χ^2^ value of 1.89 (Figure 2A). Cα models were generated for 33 residues of NTR. Models with the NTR folded into the protein core structure were rejected. Figure 2B shows multiple possible conformations of the NTR as generated from EOM

### Thermofluor assay

**Figure 3.**
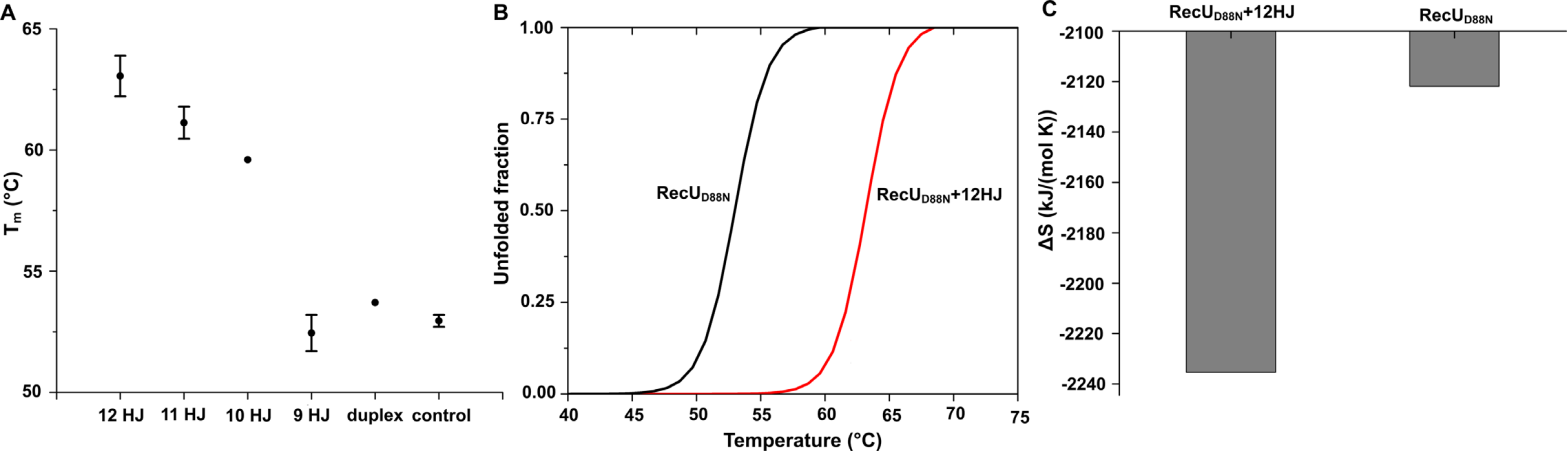
Thermofluor studies. (**A**) Thermofluor experiments were carried out to study the induced stabilization of RecU on its binding to different lengths of HJ and DNA duplex. Holliday junctions formed by the oligomers of lengths 9 bp, 10 bp, 11 bp, and 12 bp were used. DNA duplex used is same as that of the crystal structure presented here. RecU in the absence of any DNA was used as control. Thermofluor experiments for each of these systems were carried out in quadruplets. *T*_m_ was calculated from first derivatives of fluorescence intensities with respect to temperature. The scatter plot gives a clear picture of stabilization of RecU on HJ binding. (**B**) Unfolded fractions as a function of temperature for RecU_D88N_ and its complex with HJ of an arm length of 12 bp. (**C**) Conformational entropy calculated from the profiles in (B).

Thermofluor assays were used to assess the stability of RecU in complex with HJ substrates of various arm lengths. Thermofluor data (Supplementary Figure S5) was analysed to calculate the *T*_m_ values for RecU_D88N_ and its complexes with duplex (same as in the structure presented here), HJs with oligomer lengths 9, 10, 11 and 12 bp (Figure 3A). Difference in *T*_m_ value due to the binding of duplex is 0.75°C with respect unbound RecU_D88N_. For junctions of lengths 9, 10, 11 and 12 bp the change is –0.5, 6.7, 8.2 and 10.1°C, respectively. Further, the unfolding fraction as a function of the temperature (Figure 3B) was derived from thermofluor data for the RecU_D88N_ and RecU_D88N-HJ_ complex (oligomeric length of 12 bp). Based on this we calculated the configurational entropy (Δ*S*) (Figure 3C). It is seen that on interaction of RecU_D88N_ with HJ the Δ*S* is reduced indicating the stabilization of RecU in the conformational space.

### Molecular dynamics

Our experimental data from X-ray and SAXS provided a picture of a conformationally flexible NTR region and the Thermofluor assays showed that on binding to HJ, stability of RecU_D88N_ is increased. To obtain further insights into the solution dynamics of RecU in unbond and HJ bound complex, we carried out MD simulations for RecU_WT_, RecU_HJ_, RecU_ΔNTR_ and RecU_ΔNTR-HJ_. For each protein system three independent simulations, each 250 ns long, with different starting velocities were performed.

### Protein flexibility

To understand the flexibility of the protein and the effect of binding of HJ on RecU, we calculated the root mean square fluctuation (RMSF) on a per residue basis (Supplementary Figure S6) using only the Cα atoms. This analysis shows that the stalk and NTR regions are more flexible as compared to the rest of the protein in all the four systems, with the latter being much more flexible. The average RMSF and the standard error values calculated over the entire stalk region residues (NTR region was excluded from this calculation because of its high flexibility) in RecU_WT_ (0.12 ± 0.009 nm) and RecU_HJ_ (0.07 ± 0.003 nm), suggest that on binding to HJ, stalk region becomes less flexible.

### Comparison of RecU_Wt_ MD simulation ensemble with SAXS data

To check whether the structures sampled in the MD simulations are identical to the solution SAXS profile, we clustered structures from the RecU_WT_ ensemble and clustered the structures based on the backbone RMSD of the NTR region. We used the Jarvis–Patrick algorithm (62) implemented in the g_cluster tool of GROMACS with a backbone RMSD cut-off of 0.2 nm and a minimum number of 10 neighbors required to form a cluster. Simulated SAXS profiles were generated using the WAXSIS server (63) and representative structures of the top 10 populated clusters. Supplementary Table ST3 summarizes the results of the simulated SAXS profile calculation. Radius of gyration (*R*_g_) values from the RecU_WT_ simulation (25 Å) are in good agreement with the experimentally calculated value of 24.3 Å. The average chi value for the fit between experimental data and simulated SAXS profiles is 0.4 on the log scale.

### Principal component analysis (PCA)

**Figure 4.**
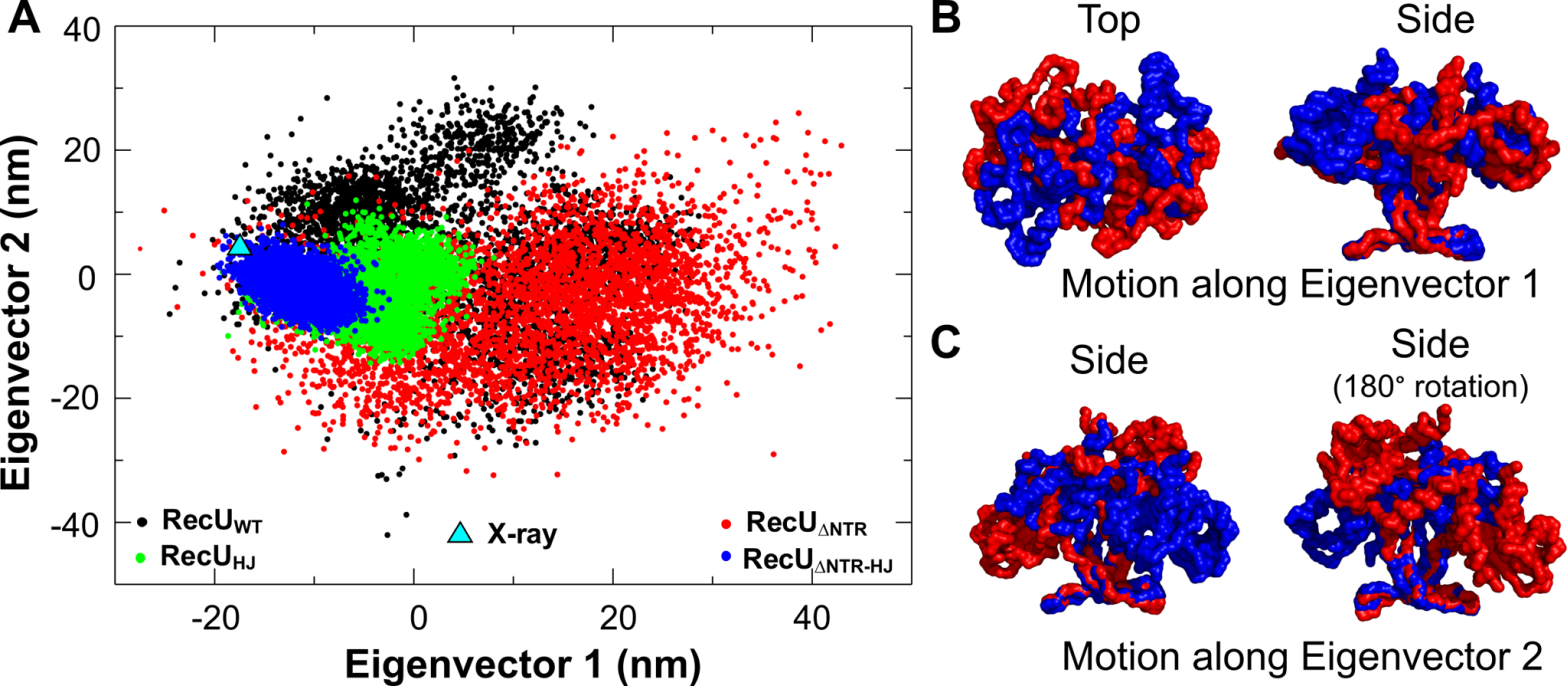
Principal Component analysis. (**A**) Projection of wild type RecU (RecU_WT_: shown in black), ΔNTR mutant RecU (RecU_ΔNTR_: shown in red) and their complexes with HJ i.e. (RecU_HJ_: shown in green), (RecU_ΔNTR-HJ_: shown in blue) along the first two eigenvectors. Sampling along both these eigenvectors is restricted in the presence of HJ. (**B**) Motion along eigenvector 1 showing rotation of mushroom cap with respect to the the stalk region. Two extreme structures along the direction of the eigenvector are shown in red and blue respectively in a surface representation of a backbone trace. (**C**) Motion along eigenvector 2 showing rocking of mushroom cap with respect to the stalk region.

To analyze domain dynamics of RecU, using the g_covar tool from GROMACS package, we extracted the eigenvectors from the three concatenated trajectories of RecU_WT_ using only the backbone atoms. RecU_HJ_, RecU_ΔNTR_, and RecU_ΔNTR-HJ_ simulations were projected onto the eigenvectors obtained from the RecU_WT_ simulations (Figure 4A). The first five eigenvectors account for 95% of the protein motion, with the first and second eigenvectors (Ev1 and Ev2) accounting for 43% and 41%, respectively. The third and fourth eigenvectors account for 8% and 3% of the protein motion, and were not analyzed further. Ev1 captured the rotation of the mushroom cap domain with respect to the stalk region (Figure 4B and Movie M1). Ev2 described the rocking motion of the mushroom cap relative to the stalk (Figure 4C and Movie M2). This results in an alternate opening and closing of the two active sites. In the open state, the maximum distance between the Cα atoms of residue R70 (tip of the stalk region) and D88 (active site) is ∼32 Å, while in the closed state the minimum distance is ∼18 Å (Supplementary Figure S7–S9). RecU_HJ_ and RecU_ΔNTR-HJ_ show restriction of domain motion along both Ev1 and Ev2. On closer inspection, projection data shows conformational selection of domain motion of RecU on its interaction with HJ, i.e. conformations which are sampled by both RecU_HJ_ and RecU_ΔNTR-HJ_ are scarcely sampled by RecU_WT_ and RecU_ΔNTR_ in the absence of HJ.

### Active site

**Figure 5.**
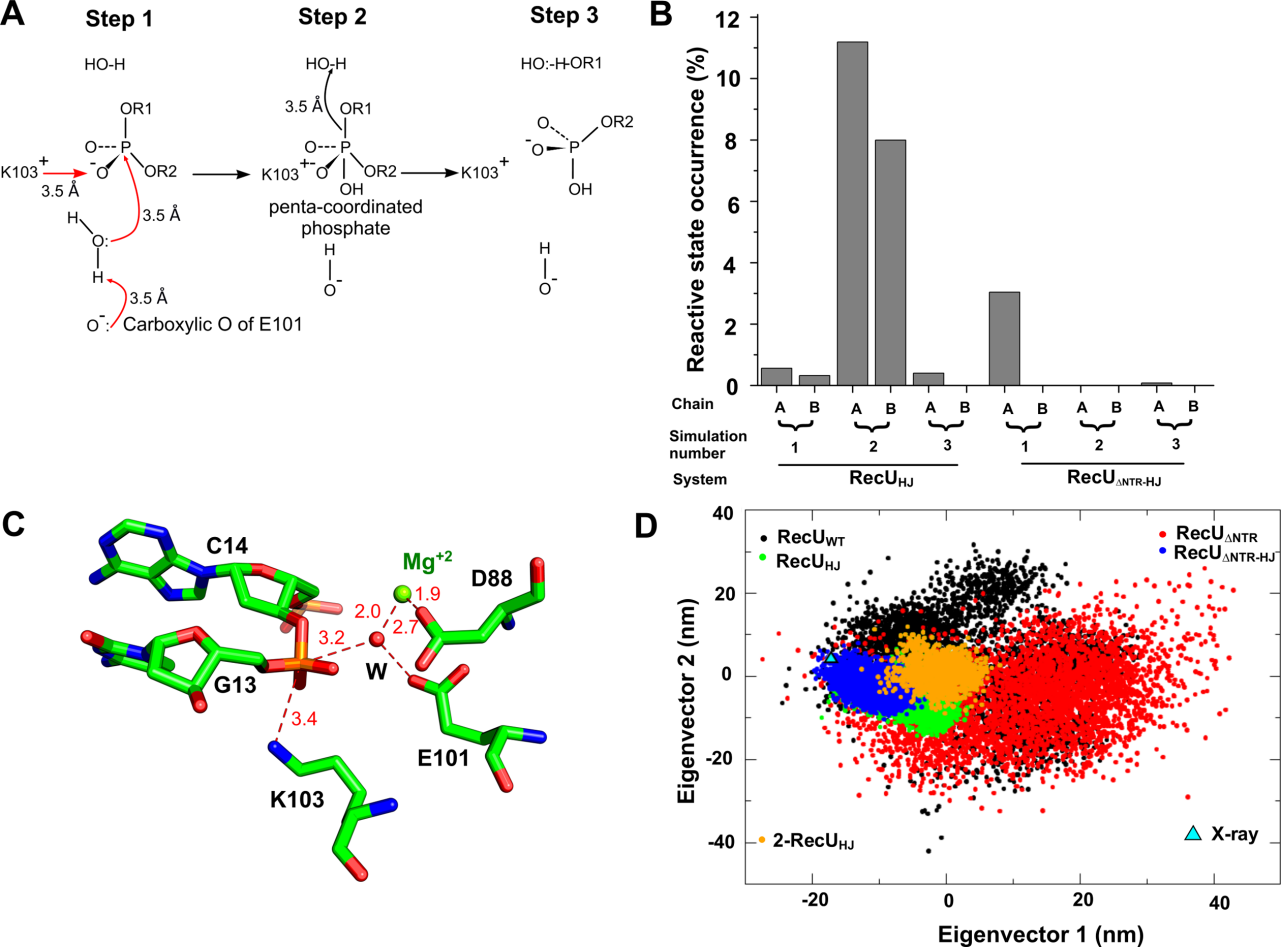
Mechanism of cleavage of phosphodiester bond at the RecU active site. (**A**) Plausible mechanism of DNA cleavage by RecU based on the mechanism reported earlier (64). Mg^+2^ will cause polarization of water molecules around it. Carboxylic oxygen of E101 (one that is not coordinated with Mg^+2^) can deprotonate a water coordinating with Mg^+2^. The deprotonated water will then attack the phosphate giving rise to penta-coordinated phosphate intermediate that is stabilized by Lewis acid K103. Another Mg^+2^ coordinated water can then act as general acid by protonating the leaving anion. (**B**) Percentage occurrence of step 1 of the above mechanism observed in the simulation of RecU in complex with holliday junction (HJ) (RecU_HJ_) and ΔNTR mutant in complex with HJ (RecU_ΔNTR-HJ_). (**C**) Atomic model of step 1 of proposed mechanism as observed in simulation (one such snapshot). DNA and active site residues are shown in stick representation. Water and Mg^+2^ are shown as spheres and are coloured in red and green respectively. (**D**) Projection of simulation-2 of RecU_HJ_ (for which the occurrence of step 1 of the proposed mechanism is highest) along eigenvector 1 and 2. RecU_WT_: shown in black; RecU_ΔNTR_: shown in red; RecU_HJ_: shown in green; RecU_ΔNTR-HJ_: shown in blue; simulation 2 of RecU_HJ_: shown in orange; projection of X-ray structure is shown as a triangle.

A possible mechanism of phosphodiester bond cleavage has been proposed earlier (64) (see Figure 5A) and is as follows: Step 1: E101 deprotonates one of the coordinating waters of an Mg^2+^ ion. Step 2: This water then carries out a nucleophilic attack onto the phosphodiester bond, resulting in a pentavalent phosphate. This pentavalent phosphate is stabilized by K103. Step 3: A second water would act as a general acid via proton donation to the leaving anion which leads to the breakage of the phosphodiester bond.

To check whether in our simulations of RecU_HJ_ and RecU_ΔNTR-HJ_, the active site of the protein adopts the reactive conformation, we counted the number of instances when the reactive conformation is sampled. We defined reactive conformation as a state when a water molecule is coordinating with Mg^2+^ and the same water molecule is within 3.3 Å of E101 (carboxylate oxygens) and within 3.3 Å of phosphate in the DNA. Figure 5B shows the percentage of simulation time that the reactive conformation is sampled in each chain for both RecU_HJ_ and RecU_ΔNTR-HJ_. This percentage ranges from 0.3% to 11% in RecU_HJ_ simulations and 0.08-3% in RecU_ΔNTR-HJ_ simulations. Figure 5C shows molecular details of one such occurrence of the reactive state. It must be noted that the statistics of reactive state occurrence have not converged across the simulations of RecU_HJ_ and RecU_ΔNTR-HJ_ and are require further investigation via longer simulations. Since the second simulation of the RecU_HJ_ complex sampled the most number of reactive state conformations (Figure 5B), we checked the projection of this particular simulation along Ev1 and Ev2. Simulations of RecU_ΔNTR-HJ_ do not sample the same conformational space sampled by the second simulation of RecU_HJ_ complex, suggesting that this simulation might have accessed the conformational space required for the reactive state formation. No such exclusive sampling was observed along Ev3 and Ev4 indicating the importance of domain dynamics observed along Ev1 and Ev2 in the functioning of RecU.

To identify the motion of RecU that results in the formation of step 1 of the active site, we performed Functional Mode Analysis (FMA) (65) on the second simulation of RecU_HJ_. FMA can be used to isolate the collective motions of a protein that describe an observable of interest, in this case, the formation of the reactive state in the active site We defined reactive state as per that of Figure 5A, i.e. if there is a water (within 3.3 Å. of Mg^2+^ and (OE1 or OE2 of E101)) and (within 3.3 Å. of phosphate atom from the DNA nucleotide). We used the first 25 eigenvectors from the PCA as they capture ∼99% motion of the protein. FMA was performed independently for chain A and B, as the occurrence of step 1 is independent in each chain with correlation coefficient of 0.035. It is observed that the formation of step 1 results from the breathing motion of the mushroom cap.

## DISCUSSION

HJ resolvases are specific nucleases which nick the four arms of an HJ at the end of the recombination process. RecU is structurally different from the other HJ resolvases in the family, because of the presence of a central stalk region (20). Though previous biochemical reports elucidated the role of this stalk region (4) and a 33-residue NTR (24) in the activity of RecU, the structural information on a RecU–HJ complex and its dynamics has been limited. Even though a number of resolvase–HJ complex structures are known (11), no HJ complexed structures have been solved from RecU.

Here, we present the first structure of a RecU complex with a bound 12 base palindromic dsDNA fragment, a visible NTR and stalk regions determined at 3.2 Å resolution. Although the low resolution limits the information on the molecular details of interface interactions, we are still clearly able to determine the positioning of the dsDNA. This structure with DNA allowed us to model a bound HJ, was used to carry out MD simulations and revolutionize our understanding of the conformational dynamics of RecU–HJ complex formation. Based on the crystal structure of the RecU–DNA complex, the binding of HJ to RecU could be driven by hydrogen bonds as well as non-bonding interactions. Two hydrogen bonds between K165 and S166 of RecU and the phosphate oxygens of DNA are observed in all four chains. Further, we also observed that F81 is in a position to form a flat hydrophobic interface for binding of HJ, similar to that seen in RuvC (66). Mutational analysis show that a RecU–F81A mutant poorly recognizes and distorts HJs (4).

The current crystal structure gives the molecular detail of the stalk region which was unobserved in the electron density maps of the previously reported crystal structure of *B. subtilis* RecU (20). The structure of a RecU homologue from *B. stearothermophilus* (22) shows a stalk region structure stabilized by crystal contacts, whereas in the crystal structure described here, it is stabilized by its interaction with DNA. Further, the arrangement of the main body or mushroom cap region of the enzyme with respect to the stalk region is asymmetric in the structure of *B. stearothermophilus* RecU, unlike the symmetric arrangement in the crystal structure of RecU–DNA complex presented here. This supports the notion that the mushroom cap and stalk region have conformational flexibility. RMSF analysis of our MD simulations confirm the flexibility of the stalk region and when RecU is bound to HJ, there is a decrease in flexibility.

Restricted sampling along Ev1 and Ev2 is observed when RecU is bound to HJ. This is in agreement with the thermofluor studies on complexes of RecU with HJ of varying arm lengths which showed effective stabilization of RecU on binding to HJ. Furthermore, the conformational entropy for RecU alone is more than that of its complex with HJ, indicating restrictive sampling of conformational subspace of RecU upon with HJ. Our results are in agreement with the observations from earlier biochemical studies (4) that point mutation in the stalk region results in decreased RecU cleavage activity, as the stalk region in coordination with mushroom cap is required for the domain dynamics.

Similar to this stalk region, the 33 residue long NTR has been shown to be important for the binding of RecU to the HJ (24). It has been unobserved in previous X-ray structures of RecU (20,64) owing to its high flexibility as also noted in our SAXS analysis. In the crystal structure reported here, this region could be modeled completely in one of four chains (chain D) and could be partially modeled in two other chains. In chain D, the presence of the NTR in the electron density is the result of crystal contacts and we confirmed this using composite omit maps and NCONT analysis.

Conformations of this NTR calculated based on the EOM indicates that this region can exist in multiple conformations in solution. It should also be noted that the conformation of NTR as observed in the chain D of crystal structure is not seen in the NTR ensemble generated by EOM. MD simulations with the NTR region (RecU_WT_ and RecU_HJ_) were started from the chain-D NTR conformation. Specifically, the conformations of RecU_WT_ simulations can be compared with the RecU_D88N_ SAXS data and the agreement between them implies that the MD simulations started from X-ray structure have moved into the conformational space in solution.

Earlier studies have further reported that the NTR deletion mutants have low activity (24). MD simulations of RecU_ΔNTR_-_HJ_ complex allowed us to understand this effect of deletion of the NTR on the activity of RecU by checking the occurrence rate of a reactive conformation in the active site. A RecU_ΔNTR_-_HJ_ complex samples the reactive state of the active site only 3% of the time in contrast to 20% of the time in a RecU_HJ_ complex ensemble. The deleterious effect of a ΔNTR mutant is also evident from the projection of MD trajectories along Ev1 and Ev2. The RecU_ΔNTR_-_HJ_ complex scarcely samples in the region sampled by a simulation (simulation-2) RecU_HJ_, which the highest occurrence of reactive states in the active site. This is in agreement with experimental observations that a ΔNTR mutant of RecU shows less activity as compared to RecU_WT_ (24).

Interestingly, the conformational sampling seen in simulation-2 of RecU_HJ_ is exclusive and the same conformational space is scarcely sampled by simulation-1 and simulation-3 of RecU_HJ_, suggesting conformational selection in the formation of reactive state (i.e. step 1 in the proposed mechanism). Formation of the reactive state in each active site is independent of the second active site in agreement with earlier reports on RuvC supporting sequential mode of cleavage (67). As MD simulations do not support bond breaking or making, the effect of the formation of a reactive state in one active site, on the cleavage in the other active site could not be studied. As deduced from FMA, the breathing motion of RecU mushroom cap facilitates in the formation of the reactive state. Though this motion is symmetric, reactive state formation in one active site is independent of the other.

**Figure 6.**
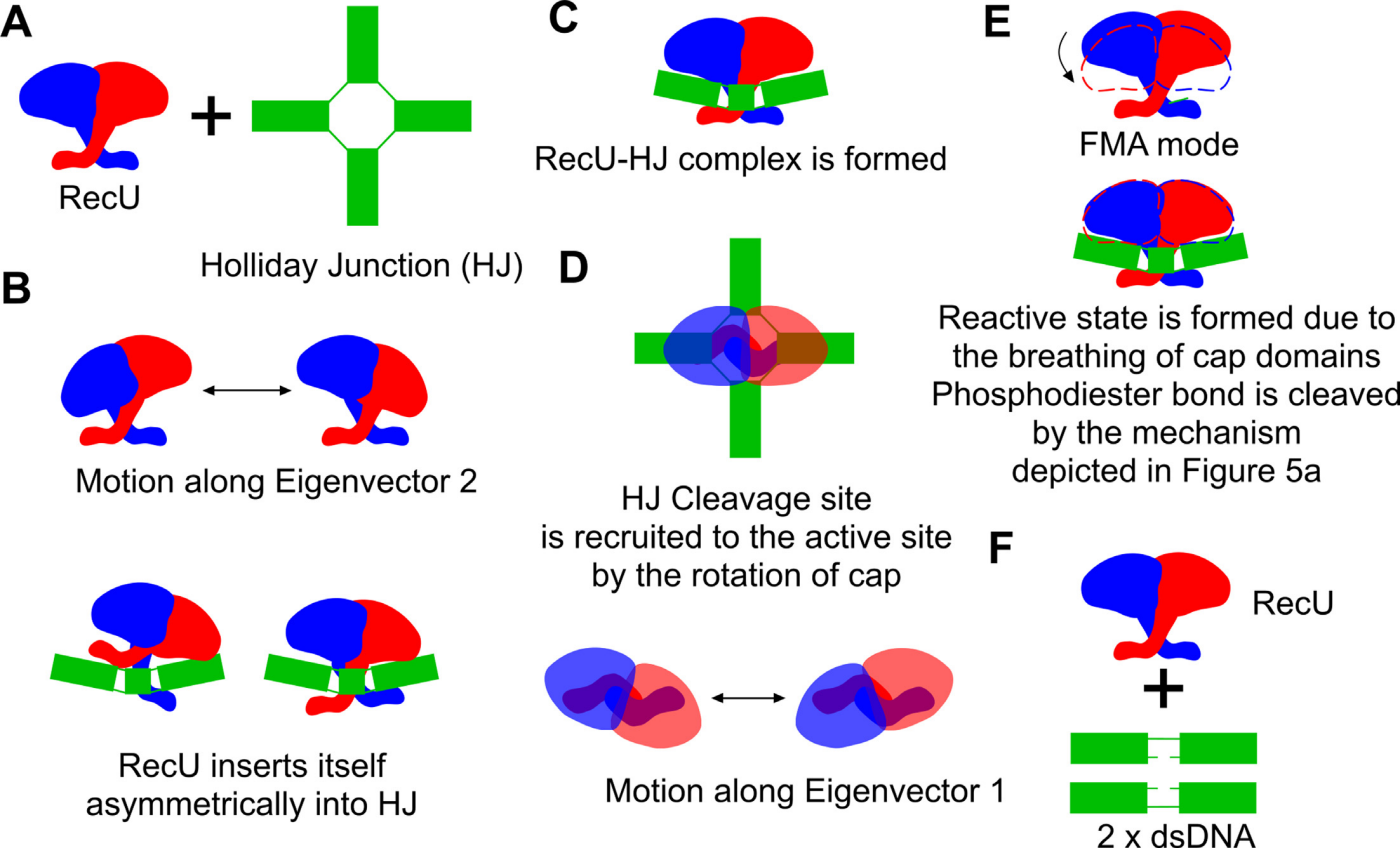
Plausible mechanism of binding and insertion of RecU in HJ. (**A**) RecU and a Holliday junction (HJ). (**B**) RecU in the unbound form exhibits rocking motion as described by the Ev2. With this domain motion RecU inserts itself in the HJ. (**C**) RecU-HJ complex is formed. (**D**) RecU then searches for the cleavage site by the rotation of the cap domain (motion observed along eigenvector Ev1). (**E**) Reactive state in the active site is then formed due to the breathing motion of mushroom cap. This breathing motion was observed in the functional mode analysis on the occurrence of the reactive state. Once reactive state is formed, phosphodiester bond cleavage occurs. (**F**) Cleaved HJ then diffuses from the RecU.

Based on asymmetric and symmetric X-ray structures of mushroom cap with respect to the stalk region from *B. stearothermophilus* and *B. subtilis* respectively, SAXS analysis, thermofluor data and extensive MD simulations we propose the following mechanism of binding of RecU and its action on HJ (Figure 6). The rocking motion of the mushroom cap with respect to the stalk region plays a role in the insertion of a RecU dimer into the HJ (Figure 6). Then the rotation of the mushroom cap with respect to the stalk region aid RecU to search and locate the cleavage site. Further, NTR might play a role in the stabilization of the reactive state. Phosphodiester bond is cleaved by the mechanism proposed earlier (64) involving nucleophilic attack of water onto the backbone phosphate of DNA. A high resolution structure of RecU-HJ complex and reaction intermediate along with the hybrid quantum mechanics/molecular mechanics calculations might allow us to get more insights into dynamics of reactive state formation and the complete reaction mechanism.
